# Flow measurement at the aortic root - impact of location of through-plane phase contrast velocity mapping

**DOI:** 10.1186/s12968-016-0277-7

**Published:** 2016-09-07

**Authors:** Litten Bertelsen, Jesper Hastrup Svendsen, Lars Køber, Ketil Haugan, Søren Højberg, Carsten Thomsen, Niels Vejlstrup

**Affiliations:** 1 Department of Cardiology, The Heart Centre, Rigshospitalet, University of Copenhagen, Copenhagen, Denmark; 2 Department of Radiology, The Diagnostic Centre, Rigshospitalet, University of Copenhagen, Copenhagen, Denmark; 3 Danish National Research Foundation Centre for Cardiac Arrhythmia (DARC), Copenhagen, Denmark; 4 Department of Clinical Medicine, Faculty of Health and Medical Sciences, University of Copenhagen, Copenhagen, Denmark; 5 Department of Cardiology, Roskilde Hospital, Roskilde, Denmark; 6 Department of Cardiology Y, Bispebjerg Hospital, Copenhagen, Denmark

**Keywords:** Cardiovascular magnetic resonance, Flow, Through-plane phase contrast velocity mapping, Volumetry, Sinotubular junction, Mitral regurgitation

## Abstract

**Background:**

Cardiovascular magnetic resonance (CMR) is considered the gold standard of cardiac volumetric measurements. Flow in the aortic root is often measured at the sinotubular junction, even though placing the slice just above valve level may be more precise. It is unknown how much flow measurements vary at different levels in the aortic root and which level corresponds best to left ventricle volumetry.

**Methods:**

All patients were older than 70 years presenting with at least one of the following diagnoses: diabetes, hypertension, prior stroke and/or heart failure. Patients with arrhythmias during CMR and aortic stenosis were excluded from the analyses.

Stroke volumes were measured volumetrically (SV_ref_) from steady-state free precision short axis images covering the entire left ventricle, excluding the papillary muscles and including the left ventricular outflow tract. Flow sequences (through-plane phase contrast velocity mapping) were obtained at valve level (SV_V_) and at the sinotubular junction (SV_ST_).

Firstly, SV_V_ and SV_ST_ were compared to each other and secondly, after excluding patients with mitral regurgitations to ensure that stroke volumes measured volumetrically would theoretically be equal to flow measurements, SV_V_ and SV_ST_ were compared to SV_ref_.

**Results:**

Initially, 152 patients were included. 22 were excluded because of arrhythmias during scans and 9 were excluded for aortic stenosis. Accordingly, data from 121 patients were analysed and of these 63 had visually evident mitral regurgitation on cine images.

On average, stroke volumes measured with flow at the sinotubular junction was 13–16 % lower than when measured at valve level (70.0 mL ±13.8 vs. 81.8 mL ±15.5). This was in excess of the expected difference caused by the outflow to the coronary arteries.

In the 58 patients with no valvulopathy, stroke volumes measured at valve level (79.0 mL ±12.4) was closest to the volumetric measurement (85.4 mL ±12.0) but still significantly lower (*p* < 0.001). Flow measured at the ST-junction (68.1 mL ±11.6) was significantly lower than at valve level and the volumetric measurements. The mean difference between SV_ref_–SV_V_ (6.4 mL) and SV_ref_-SV_ST_ (18.2 mL) showed similar variances (SD 7.4 vs. 8.1 respectively) and hence equal accuracy.

**Conclusions:**

Aortic flow measured at valve level corresponded best with volumetric measurements and on average flow measured at the sinotubular junction underestimated flow approximately 15 % compared to valve level.

**Trial registration:**

ClinicalTrials.gov identifier: NCT02036450. Registered 08/01/2014.

## Background

**Fig. 1:**
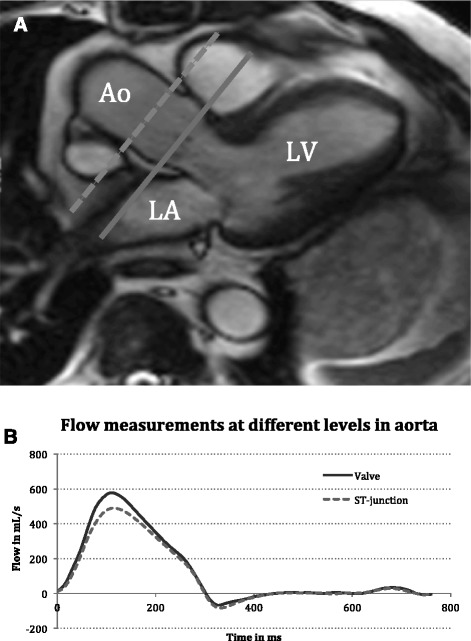
Example of flow measurements in a patient. Panel **a** illustrates placement of imaging planes at the ST-junction (dashed line) and at valve level (solid line). Panel **b** shows the resulting flow curves for the two different imaging planes. *Ao*  aorta, *LV*  left ventricle, *LA*  left atrium,  *ST*  sinotubular, *mL*  millilitres, *s*  seconds, *ms*  milliseconds

Cardiovascular magnetic resonance (CMR) is considered the gold standard for cardiac volumetric measurements [1]. The most widespread method for measuring flow with CMR is free-breathing through-plane phase-contrast velocity mapping. In general, aortic flow is often measured at the sinotubular (ST) junction [2, 3] even though placing the slice at the tip of the valve cusps in systole may be more precise [4, 5]. An exception to this is in case of aortic stenosis where it is recommended to do phase velocity encoding at valve level to avoid the disturbed flow further downstream as the jet breaks down [2, 6]. The work by Chatzimavroudis et al. argue that regurgitant flow is more precise when measured at valve level [5] but we have been unable to detect any research illuminating which level is more precise with regards to forward flow. Flow measured at different levels in the aorta is used equally in the clinic and the literature provides examples of papers where both levels of measurement are used [7, 8], but in our experience, they are by no means equal when compared (see Fig. 1 for example). This difference could to some extend be explained by the fact that the coronary arteries originate between the two imaging planes. The coronary blood flow is normally estimated to represent 5 % of the cardiac output [9] and would hence explain such a difference.

**Fig. 2:**
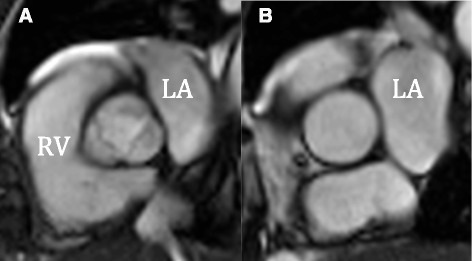
Slices at valve (**a**) and ST-junction (**b**). Flow is quickly measured at the ST-junction because of the round lumen, but turbulence and acceleration makes the flow complex and the slice is above the coronary arteries. At valve level the flow is more laminar, which makes velocity encoding more reliable but because of the shape manual analyses are often necessary. *ST* sinotubular, *RV* right ventricle, *LA* left atrium

The difference between the two slices can be appreciated in the images in Fig. 2. The lumen at the ST-junction is round which is easily measured using automatic software. The lumen at valve level is shaped like a three leaf clover making delineation more difficult and this most often requires manual tracing, which is much more time consuming than automatic analyses.

We wished firstly to quantify how much the flow measurements vary at different levels in the aortic root and secondly to investigate which level corresponds best with the gold standard, left ventricle volumetry. We chose a group of patients with multiple morbidities (see Methods) to make results applicable to a broad population of patients.

## Methods

### Study design and participants

All participants were older than 70 years and presented with at least one of the following diagnoses: Diabetes, hypertension, prior stroke and/or heart failure. Patients with arrhythmias during CMR were excluded from further analyses since arrhythmias make flow measurements unreliable [4]. Patients with aortic stenosis (AS) were excluded from the main analyses due to the difficulties in acquiring reliable flow results from the complex turbulent flow surrounding the high velocity jet caused by the stenosis [2, 10, 11]. We performed a post-hoc exploratory flow analysis in the subgroup of patients with AS.

To assess whether patients had mitral regurgitations (MR) all accessible SSFP images were studied visually for a signal loss in the left atrium in front of the mitral valve. This assessment was performed without regarding flow measurements.

### Data acquisition

All scans were performed on a 1.5 Tesla Siemens Espree, Erlangen, Germany. The volumetric measurements of the left ventricle (LV) were assessed using steady-state free precession cine sequences (SSFP) (8 mm; no gap; 25 phases; field of view (300-360) x 360 mm adjusted for each patient; matrix size (174-192) x 192 voxels) obtained at 7 to 10 s end-expiratory breath-holds. Short axis images covering the entire LV were obtained. Long axis images, including two-chamber, three-chamber and four-chamber views, were acquired for planning of the short axis images and to aid in delineation of the ventricle. Furthermore, for positioning of flow planes, two orthogonal views of the left ventricular outflow tract were acquired.

Flow sequences (free-breathing through-plane phase-contrast non-navigator-gated sequences) were obtained at the tip of the valve cusps in systole (judged from SSFP images) and at the ST-junction. To assure perpendicularity to the aorta, the imaging plane of the flow sequence was simultaneously viewed in two orthogonal SSFP images of the left ventricular outflow tract. Flow measurements were done in the isocenter of the magnet. Images were checked for aliasing and velocity encoding adjusted if needed. The following parameters were used: 50 phases; TE = 2.8 ms; TR = 34.9; K-space segmentation factor 4; field of view (240-320) x 320 mm adjusted for each patient; matrix size (192-256) x 256 voxels; pixel spacing 1.25 x 1.25 mm; slice thickness = 5 mm; temporal resolution was 12-26 ms. flip angle 30°; 3 averages; velocity encoding of 200 cm/s and increased if aliasing present; acquisition time 1.14-2.26 min.

### Data analysis

Dedicated software (CVI42 v. 5.1.0, Circle Cardiovascular Imaging Inc., Calgary, Canada) was used for post-processing.

LV end-systolic and end-diastolic phases were identified and traced manually at the endocardial border according to the LV blood pool area, excluding the papillary muscles from the LV cavity and including the left ventricular outflow tract as part of the LV cavity [12]. Endocardial borders were delineated using windowing, and the trabeculation was excluded from the blood pool. Left ventricular epicardium was segmented to compare myocardial mass in end-diastole and end-systole to avoid overestimation of end-systolic volume.

Aorta was traced semi-automated with manual correction. Background correction, consisting of a ROI in the muscle and fat in the anterior thoracic wall, was used on all images. For the valve level measurements the entire aortic area was included (the three leaf clover *–* see Fig. 2).

The stroke volumes measured with flow at valve level (SV_V_), at the ST-junction (SV_ST_) and the volumetrically measured SV from SSFP sequences (SV_ref_) were registered.

### Statistics

Paired students t-tests were used for comparisons. Two comparisons were made:

- The two flow measurements (SV_V_ and SV_ST_) were compared to each other.
- The two flow measurements (SV_V_ and SV_ST_) were, in turn, compared to the volumetric measurements (SV_ref_). For this second analysis, patients with mitral regurgitations were excluded to ensure that stroke volumes measured volumetrically would theoretically equal flow measurements, i.e. only reflecting forward aortic flow.

Bland-Altman plots were used to visualize the difference and variability between two measurements.

Interobserver variability was studied by separate blinded analyses of 10 randomly selected scans by two investigators. The results were assessed with regression analyses and Bland-Altman analyses and plots.

## Results

Initially, 152 patients were included in the study, 22 were excluded because of arrhythmias (multiple ventricular extra systoles, strong respiratory sinus arrhythmia) and 9 had AS. Accordingly, data from 121 patients were analyzed, and 63 showed signs of mitral regurgitations and were excluded from the second analysis (see Table 1 for demographics). The patients also had en echocardiogram performed, and these were reviewed for mitral regurgitation. The group with MR identified on echo did not match the CMR group with MR, but defining MR from echo did not change the results (data not shown).

**Table 1:** Demographics of included patients

Patients (no = 121)	Mean ± standard deviation
Mean age	76.1 ± 4.5
Male sex	70 (57.9 %)
Heart rate (beats per minute)	70.5 ± 11.3
LVEF (%)	68.0 ± 7.3
Body surface area (m^2^)	1.97 ± 0.21

### Comparison of stroke volumes according to imaging plane

Stroke volume measured at the ST-junction, SV_ST_ was 70.0 mL (SD 13.8) and at valve level, SV_V_ was 81.8 mL (SD 15.5). Hence, SV_ST_ was on average 15 % (﻿ 95 % confidence interval (CI): 13–16 %) lower than SV_V_ (*p* < 0.0001). No difference was found between males and females.

**Fig. 3:**
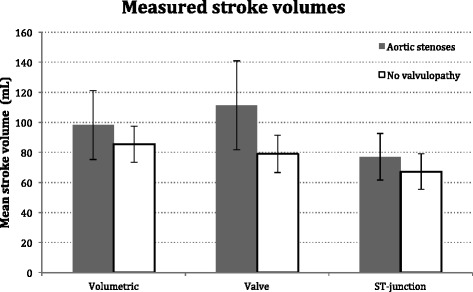
Mean stroke volumes measured volumetrically and with flow at valve level and the sinotubular (ST) junction. Patients with aortic stenoses (*n* = 9) and no valvulopathy (*n* = 58), respectively. Error bars indicate standard deviation. *mL*  millilitres

As an interesting observation, we found in the subgroup of AS patients (*n* = 9) excluded from the main analyses, SV_V_ (111.3 mL, SD 29.6) tended to overestimate the flow compared to SV_ref_ (98.1 mL, SD 23.0), as opposed to the underestimation seen in the rest of the population. SV_ST_ (77.1 mL, SD 15.5) in these patients seemed to be more reliable and corresponded better with the results of the patients with no AS (see Fig. 3).

### Comparison of stroke volumes measured with flow and volumetrically

**Fig. 4:**
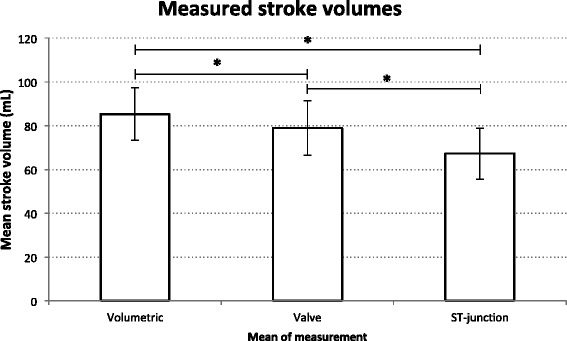
Mean stroke volumes measured volumetrically and with flow at valve level and the sinotubular (ST) junction. *N* = 58, patients with mitral regurgitations excluded. Error bars indicate standard deviation. *mL*  millilitres. * indicate statistically significant differences (*p* < 0.0001)

In the 58 patients with no valvulopathy (see Table 2 and Fig. 4), stroke volumes measured at valve level (79.0 mL, SD 12.4) was closest to the volumetric measurement (85.3 mL, SD 12.1) but still significantly different (*p* < 0.0001) and SV_ref_ was on average 8 % (CI: 6–11 %) higher than SV_V_. SV measured with flow at the ST-junction (68.1 mL, SD 11.6) was significantly lower than at valve level (*p* < 0.0001) and the volumetric measurements and SV_ref_ was on average 28 % (CI: 24–32 %) higher than SV_ST_.

**Table 2:** Stroke volumes measured volumetrically (SV_ref_) and with flow at valve level (SV_V_) and the ST-junction (SV_ST_). *ST* sinotubular, *mL* millilitres, *MR* mitral regurgitation, *SD* standard deviation

Stroke volumes in mL	All patients	MRs excluded
	*N* = 121	*N* = 58
	Mean (1SD)	Mean (1SD)
SV_ref_	94.6 (18.3)	85.3 (12.1)
SV_V_	81.8 (15.5)	79.0 (12.4)
SV_ST_	70.0 (13.8)	67.2 (11.8)
SV_ref_- SV_V_	12.8 (10.3)	6.3 (7.7)
SV_ref_- SV_ST_	24.6 (10.6)	18.1 (8.7)

**Fig. 5:**
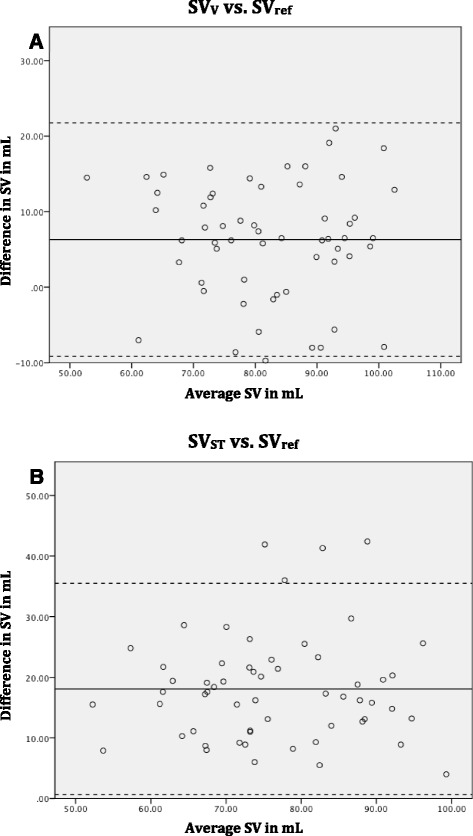
Bland Altman plot. The continuous line represents the mean (bias) and the dotted lines represent the 95 % limits of agreement for **a** Stroke volume measured with flow at valve level (SV_V_) compared to volumetric method (SV_ref_) **b** Stroke volume measured with flow at the sinotubular junction (SV_ST_) compared to volumetric method (SV_ref_). *SV*  stroke volume, *mL*  millilitres

The mean differences between Sv_ref_–SV_V_ (6.3 mL) and SV_ref_-SV_ST_ (18.1 mL) showed similar variances (SD 7.7 vs. 8.7 respectively). Bias and limits of agreement were assessed by Bland-Altman analyses (see Fig. 5).

The time it took to analyze each flow measurement was not recorded, but it does take substantially longer at valve level where many ROIs needs to be hand drawn.

**Fig. 6:**
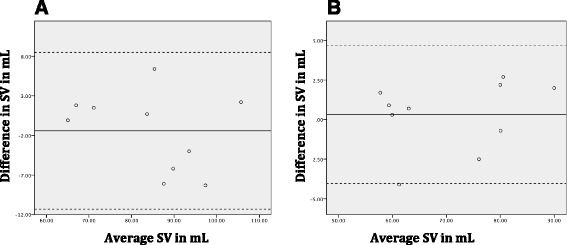
Bland-Altman plots of interobserver variability. The continuous line represents the mean (bias) and the dotted lines represent the 95 % limits of agreement for **a** Stroke volume measured with flow at valve level **b** Stroke volume measured with flow at the sinotubular junction. Bland-Altman analyses showed no proportional bias (valve *p* = 0.35, ST *p* = 0.44). *SV*  stroke volume, *mL* milliliters

Linear regression analyses of the interobserver variability showed correlations between the two investigators of 94 % for valve measurements and 98 % for ST-junction measurements. Bland-Altman Plots for the two investigators results can be seen in Fig. 6. Bland-Altman analyses showed no proportional bias.

## Discussion

In the present study, we measured flow with free-breathing through-plane phase-contrast velocity mapping at two different levels in aorta, the ST-junction and at valve level. We compared the two flow measurements to each other and in turn with the gold standard, left ventricle volumetric measurements. We found that flow measured in aorta is highly dependent on the location of the slice in the aortic root. Our results indicate that measuring flow at the ST-junction underestimates flow compared both to volumetric measurements and flow at valve level. If flow measured at the ST-junction is subsequently used to estimate for example mitral regurgitations this could result in a systematic overestimation. Our results indicate a 13–16 % underestimation of aortic flow when measured at the ST-junction compared to valve level. Even though the coronary ostia are situated between the two planes, the coronary blood flow only comprises approximately 5 % of the total cardiac output [9], e.g 4.3 mL as in the case of a stroke volume of 85.4 mL.

Even though volumetry is considered gold standard, it is subject to assumptions and simplifications resulting in possible errors. Two important sources of error are the amount of trabeculation included in the blood pool, especially difficulties in segmenting trabeculations at end-systole and delineation of the basal areas of the ventricle, which comprise a large portion of the total volume.

Measuring aortic flow at valve level corresponded best with the volumetric measurements in the present study. Still, SV measured volumetrically was on average approximately 6 mL or 8 % (CI 6–11 %) higher than when measured with flow at valve level. When comparing results obtained with different methods, as we have done in this study, it is important to keep in mind under which conditions these were acquired. During flow measurements the patients are instructed to breathe normally, however for SSFP cine sequences the patients are instructed to hold their breath during end-expiration of a normal breathing pattern. It is essential that breath is held at a normal breathing pattern, since a large lung volume will result in decreased SV due decreased venous return to the heart [13]. Van den Hout et al. have found that SV decreased during inspiration and increased during expiration in healthy subjects [14]. This was in the level of approximately 7 mL and may provide an explanation for the difference we found between volumetric measurements and flow measurements. Since breath-hold data acquisition has been demonstrated to be accurate and reliable [15] this difference is likely due to patients not complying completely with instructions of breath-hold at normal end-expiration breathing pattern, hence performing a slight Valsalva manoeuvre and increasing their cardiac output slightly in the first 10 to 15 s. An alternative explanation could be that small mitral or aortic regurgitations are not visible on SSFP images. A jet of some magnitude is required to be visible on SSFP images [16]. Hence, since mitral regurgitations were determined visually, the difference between SV_ref_ and SV_V_ could be due to small missed mitral regurgitations. Through-plane velocity mapping relies upon the imaging plane being perpendicular to the flow being measured. Throughout the cardiac cycle the movements of the heart causes the aorta to move, which may cause slight angulation to the imaging plane. This angle could be different for the ST-junction and valve level, which may cause measurements to differ.

There is evidence to suggest that the myocardial blood flow is increased in the elderly compared to a younger population [17]. This increase can account for some of the here described difference but in our opinion, it is unlikely that the coronary blood flow in the elderly accounts for 15 % of the total cardiac output.

As an interesting observation, we saw that in the relatively small number of patients with AS excluded from the main analyses, flow measurements at valve level tended to overestimate SV compared to the volumetric measurements contrasting the underestimation seen in the rest of the population (see Fig. 3). Furthermore we found that flow measured at the ST-junction seemed to correspond better to measurements in patients without AS. These observations have to be studied with the reservation that the patients on average had increased SVs presumably due to hypertrophy of the left ventricle caused by their AS. Since only a small number of patients with AS were studied, these results should be investigated further and confirmed in a larger population.

Another interesting finding was that the mean differences between SV_ref_–SV_V_ and SV_ref_-SV_ST_ showed similar variances and hence equal accuracy. So even though measuring flow at valve level may be more time consuming, we found no evidence that this method is less accurate than measuring flow at the ST-junction.

## Conclusion

When using CMR one has to be aware that results are highly dependent on several factors, including placement of the slice. Especially in patients with numerous extra systoles flow measurements are unreliable. We conclude that:

- Aortic flow measurements are highly dependent on slice position in the aortic root
- Stroke volumes measured with flow at valve level corresponded best with left ventricle volumetric measurements
- On average, flow measured at the ST-junction estimated left ventricular stroke volumes 15 % lower than stroke volumes measured with flow at valve level

## Abbreviations

AS: Aortic stenosis
CMR: Cardiovascular magnetic resonance
LV: Left ventricle
mL: Millilitres
MR: Mitral regurgitations
SD: Standard deviation
SSFP: Steady-state free precession
ST: Sinotubular
SV_ref_: Stroke volume measured volumetrically
SV_ST_: Stroke volume measured with flow at the sinotubular junction
SV_V_: Stroke volume measured with flow at valve level
